# Modification of Gold Zeolitic Supports for Catalytic Oxidation of Glucose to Gluconic Acid

**DOI:** 10.3390/ma14185250

**Published:** 2021-09-13

**Authors:** Adrian Walkowiak, Joanna Wolska, Anna Wojtaszek-Gurdak, Izabela Sobczak, Lukasz Wolski, Maria Ziolek

**Affiliations:** Faculty of Chemistry, Adam Mickiewicz University, Uniwersytetu Poznańskiego 8, 61–614 Poznań, Poland; anna.gurdak@amu.edu.pl (A.W.-G.); sobiza@amu.edu.pl (I.S.); wolski.lukasz@amu.edu.pl (L.W.); ziolek@amu.edu.pl (M.Z.)

**Keywords:** gold zeolites, amino-organosilane modifier, boron modifier, selective glucose oxidation with O_2_ and H_2_O_2_, microwave-assisted oxidation, base-free oxidation

## Abstract

Activity of gold supported catalysts strongly depends on the type and composition of support, which determine the size of Au nanoparticles (Au NPs), gold-support interaction influencing gold properties, interaction with the reactants and, in this way, the reaction pathway. The aim of this study was to use two types of zeolites: the three dimensional HBeta and the layered two-dimensional MCM-36 as supports for gold, and modification of their properties towards the achievement of different properties in oxidation of glucose to gluconic acid with molecular oxygen and hydrogen peroxide. Such an approach allowed establishment of relationships between the activity of gold catalysts and different parameters such as Au NPs size, electronic properties of gold, structure and acidity of the supports. The zeolites were modified with (3-aminopropyl)-trimethoxysilane (APMS), which affected the support features and Au NPs properties. Moreover, the modification of the zeolite lattice with boron was applied to change the strength of the zeolite acidity. All modifications resulted in changes in glucose conversion, while maintaining high selectivity to gluconic acid. The most important findings include the differences in the reaction steps limiting the reaction rate depending on the nature of the oxidant applied (oxygen vs. H_2_O_2_), the important role of porosity of the zeolite supports, and accumulation of negative charge on Au NPs in catalytic oxidation of glucose.

## 1. Introduction

Over 30 years of gold catalysis, since the Haruta’s pioneering discovery [1] of unique activity of Au nanoparticles (NPs), has brought a huge number of scientific publications concerning the application of Au NPs, usually supported on porous matrices, in different oxidation processes. The mechanisms of many reactions performed successfully on gold catalysts have not been fully disclosed yet. Recently, Hutchings [2] has pointed out that work on improvement of catalyst performance or designing new ones must be based on deep understanding of the reaction mechanism. In the last few years, Au NPs have been deposited on several different supports (e.g., mesoporous silica [3], metal oxides [4,5,6] and carbons [7,8]) and applied as catalysts in glucose oxidation. However, glucose oxidation carried out over gold catalysts supported on zeolites belongs to the reactions whose pathways have not been fully solved [9]. Attempts at unravelling the reaction mechanism can be undertaken on the basis of some knowledge about the structure and type of active sites. The aim of this study was to provide the necessary information on the active sites and the interaction between modifiers in the zeolites’ supports and active gold species, which is known to affect the catalytic activity.

For the catalysts based on zeolites as supports and gold NPs as the main active centers it is relatively easy to determine the structure and type of active sites (e.g., Brønsted acid sites (BAS) in zeolites). Moreover, zeolites can be easily modified by isomorphous substitution in their skeleton (e.g., by incorporation of boron instead of aluminum in the aluminosilica framework [10,11]) or by post-synthesis modification (e.g., by functionalization of the zeolite surface with amino-organosilanes [12,13]). Changes in the zeolite composition imply changes in the surface (acid-base) properties and thus modify the interaction between the support and gold NPs. Furthermore, a large number of zeolite structures [14] opens an opportunity to study the effect of the support structure on the activity and selectivity of gold catalysts. Moreover, the acid-base active centers in zeolites can act as sites of chemisorption of organic compounds applied as reactants and, therefore, take part in the mechanism of catalytic oxidation on Au NPs. Acid-base properties of non-reducible supports (such as zeolites) have been recognized as important in oxidation processes [15]. As concerns sugars oxidation, the BAS on the catalyst surface are able to protonate glucose molecules making them more susceptible to oxidation [16]. Recently [17], we have postulated that the first step of the base-free glucose oxidation on gold supported on Beta zeolite is the protonation of the carbonyl oxygen (at carbon atom C1) in the glucose molecule, in which the BAS of the zeolite support take part. It has been indicated that the chemisorption of glucose on BAS is not the rate limiting step of the reaction, but the strength of BAS determines the selectivity of the reaction. Oxygen chemisorption on Au NPs and/or its interaction with chemisorbed glucose has been postulated as limiting the total reaction rate. The role of the Au NPs size in glucose oxidation has been stressed in many papers and it has been indicated that the optimal gold particle size (to ensure the highest glucose conversion) is ca 7 nm [18] or 9 nm [9,19] for gold supported on carbons and on zeolites [17]. The Au NPs of this size have also been identified as the most active in glucose oxidation on AuPd supported on titanate nanotubes [20]. The question is, if this size of AuNPs would ensure the best performance of all gold supported catalysts applied in glucose oxidation. To answer this question two different zeolite structures (3D Beta and 2D MCM-36) were used in this study as supports of AuNPs of the size of ca 7 nm, and the obtained gold catalysts were applied in base-free glucose oxidation.

To get a deeper insight into the role of BAS in the glucose oxidation pathway, modification of the 2D zeolite support (MCM-36) with boron to change the zeolite acidity was applied. Moreover, amine groups ((3-aminopropyl)trimethoxysilane) were introduced into the zeolite supports for their modification and tailor properties of gold phase. Another problem that we would like to address in this study concerns the role of the oxidant type (molecular oxygen vs. hydrogen peroxide) on the activity of zeolite-based gold catalysts studied.

## 2. Materials and Methods

### 2.1. Materials/Compounds

The chemicals used in this work were: glucose (Sigma Aldrich, Saint Louis, MO, USA, 99.5%), chloroauric acid (HAuCl_4_∙*x*H_2_O, Sigma Aldrich, Saint Louis, MO, USA, 99.995%), (3-aminopropyl)trimethoxysilane (APMS, Sigma Aldrich, Saint Louis, MO, USA, 97%), sodium borohydride (Sigma Aldrich, >98%), boric acid (H_3_BO_3_, Sigma Aldrich, Saint Louis, MO, USA, 99.97%), toluene (Sigma Aldrich, Saint Louis, MO, USA, HPLC grade), acetonitrile (Sigma Aldrich, Saint Louis, MO, USA, HPLC grade), SiO_2_ (Ultrasil, Wesseling, Germany, 3VN, Degussa), sodium aluminate (Riedel-de Haen, Seelze, Germany, 53% of Al_2_O_3_ and 42.5% of Na_2_O), hexamethyleneimine (HMI; Sigma Aldrich, Saint Louis, MO, USA, 99%), cetyltrimethylammonium chloride aqueous solution (CTMACl; 25 wt.%, Sigma Aldrich), tetrapropylammonium hydroxide aqueous solution (TPAOH; 40%, Merck, Darmstadt, Germany), tetraethylorthosilicate (TEOS; Sigma Aldrich, Saint Louis, MO, USA, 98%), sodium hydroxide (NaOH, POCH, Gliwice, Poland, analytical grade), nitric acid (HNO_3_, CHEMPUR, Piekary Śląskie, Poland, aqueous solution (65%), analytical grade), hydrogen peroxide (30 wt.%, StanLab, Lublin, Poland), pyridine (C_5_H_5_N, Sigma Aldrich, Saint Louis, MO, USA, 99.8%), and deionized water.

Proton form of Beta zeolite (HBeta) was obtained via calcination of the commercial ammonium form of Beta (Alfa Aesar, Haverhill, MA, USA, Si/Al = 19). Calcination conditions: 15 h in 550 °C (heating rate 1 °C min^−1^).

### 2.2. Synthesis of MCM-36 Zeolite

The MCM-36 zeolite was obtained by the modification of the layered precursor MCM-22 in the two-step procedures reported in [21]. The MCM-22 zeolite was prepared according to the procedure described in [22]. The following reagents were used for the synthesis of MCM-22 zeolite: SiO_2_ sodium aluminate, deionized water, sodium hydroxide, and hexamethyleneimine (HMI) as the template. The synthesis gel had the following composition: Si/Al = 20, H_2_O/SiO_2_ = 49, Na/SiO_2_ = 0.23, HMI/SiO_2_ = 0.51. The synthesis mixture was stirred for 30 min at r.t. (room temperature) and then loaded into a Teflon-lined Parr reactor (300 mL). Hydrothermal synthesis was carried out at 150 °C for 120 h upon continuous stirring. The product was recovered by filtration, washed with deionized water, dried at 110 °C. Dried MCM-22 zeolite (with template) was mixed with CTMACland TPAOH aqueous solutions at a relative weight ratio of 1:4.6:1.16. The mixture was continuously stirred at 90 °C for 24 h. The resulting swollen material was filtered and washed with a small amount of deionized water and dried at 80 °C overnight. The next step was pillaring of dried swollen MWW zeolite. The excess of TEOS was added as a pillaring agent to the swollen MWW zeolite. The mixture was stirred and heated under reflux at 95 °C overnight. The solid was isolated by centrifugation, hydrolyzed in the centrifugation tube by adding 10–20 mL of water and stirring overnight. It was then centrifuged and dried at 60 °C overnight. Final calcination was carried out at 550 °C for 5 h.

### 2.3. Modification of MCM-36 Zeolites with Boron

B/MCM-36 catalyst was prepared by the impregnation method using an aqueous solution of H_3_BO_3_ (assumed boron loading: 1 wt.%). The heated catalyst was placed in a flask connected to a rotary vacuum evaporator and evaporated for 1 h. Next, the proper amount of boric acid solution was added and the wet material was rotated (100 rpm) at 80 °C. After evaporation, the material was dried in an oven at 80 °C for 12 h and then calcined at 550 °C for 5 h.

### 2.4. Modification with (3-Aminopropyl)trimethoxysilane (APMS)

A given zeolite (HBeta or MCM-36 or B/MCM-36) was dispersed in toluene and to this dispersion APMS was added (2.5 mL of aminosilane per 1 g of zeolite). The as-prepared mixture was refluxed for 18 h and then filtered and washed successively with toluene, deionized water and acetonitrile. The product was dried overnight at 100 °C. The so obtained materials are labelled as NH_2_/**X**, where **X** stands for the type of zeolite support (HBeta or MCM-36 or B/MCM-36).

### 2.5. Gold Catalysts Preparation

The aminosilane-modified zeolite support was dispersed in water. Then, a certain amount of the aqueous solution of chloroauric acid was added in order to obtain ca 2 wt.% of gold loading in the final product. The amount of gold precursor required was calculated relative to the weight of the zeolite support without APMS modifier. The suspension was stirred vigorously for 1 h. After stirring, the yellowish solid with incorporated gold species was separated from the colorless solution via vacuum filtration. The resulted yellowish solid was dispersed in a small amount of water and, after 15 min of vigorous stirring, the aqueous solution of sodium borohydride was added (0.1 M; molar ratio NaBH_4_:Au = 10:1). The mixture immediately turned purple and was stirred for another 20 min. The colored product was separated by vacuum filtration and next dried in 80 °C overnight. Last of all, the samples were calcined in order to remove the aminosilane modifier (calcination conditions: 4 h in 500 °C, heating rate: 2 °C min^−1^). The as-prepared samples were denoted as Au/**X**, where **X** stands for the type of zeolite support (HBeta or MCM-36 or B/MCM-36) and made the group of calcined gold catalysts.

An independent series of amine-containing materials was prepared. This time the final products were not calcined (the synthesis procedure ended with the drying step) and hence the amino-organosilane modifier was not removed. For this series of materials the amount of gold precursor required to obtain ca 2 wt.% of gold loading in the final product was calculated relative to the weight of the zeolite support containing APMS modifier. The as-prepared materials were labeled as Au/NH_2_/**X**, where **X** stands for the type of zeolite support (HBeta or MCM-36 or B/MCM-36) and made the group of non-calcined gold catalysts.

The Au/HBeta and Au/MCM-36 samples were furthermore modified with APMS following the procedure described in Section 2.4. The as-prepared materials were denoted as NH_2_/Au/**X**, where **X** stands for HBeta or MCM-36 and made the group of calcined gold catalysts modified with amino-organosilane.

### 2.6. Characterization of the Materials

X-ray powder diffraction (XRD) patterns of zeolites were obtained at r.t. on a Bruker AXS D8 Advance (Billerica, MA, USA) apparatus using CuKα radiation (λ = 0.154 nm), with a step of 0.05° in the wide-angle range (2θ = 6–60°).

The N_2_ adsorption/desorption analysis was performed at −196 °C using a Micromeritics ASAP2020 Physisorption Analyzer (Norcross, GA, USA). The samples were pre-treated in situ under vacuum at 200 °C. The surface area calculated by the BET method, external surface area and micropore volume was estimated according to the t-plot, whereas the total pore volume was calculated at relative pressure (p/p_0_) of 0.98.

Transmission electron microscopy (TEM) investigation was carried out by means of a Tecnai Osiris instrument (FEI/Thermo Fisher, Waltham, MS, USA) operating at an accelerating voltage of 200 kV. The samples for TEM characterization were placed on a lacey-carbon film supported on a Cu TEM grid. The gold particle sizes were measured with the use of ImageJ software (1.50e version). The mean Au NPs size of each sample was calculated on the basis of 200 counts.

X-ray Photoelectron Spectra (XPS) were obtained on a spectrometer equipped with monochromatic Al-Kα source emitting photons of energy of 1486.71 eV (150 W) and a hemispherical analyzer (PHOIBOS 150 MCD NAP, SPECS, Berlin, Germany) set to the pass energy of 60 eV and 20 eV for survey and regions, respectively. XPS measurement was performed in an ultrahigh vacuum (UHV) under a pressure < 5 × 10^−9^ mbar and the flood gun was on. A studied sample was deposited on a sample holder using a double-side carbon adhesive tape. Any charging that occurred during the measurements (due to incomplete neutralization of ejected surface electrons) was accounted for by rigidly shifting the entire spectrum by a distance needed to set the binding energy of the C1s, assigned to adventitious carbon, to the assumed value of 284.8 eV.

UV-vis spectra of the samples were recorded using a Varian-Cary 300 Scan UV–vis spectrophotometer (Candela, Warszawa, Poland) equipped with a diffuse-reflectance accessory. Powdered samples were placed in a cell equipped with a quartz window. The spectra were recorded in the range from 190 to 800 nm (the wavelength resolution was 0.4 nm). Spectralon was used as a reference material.

The actual gold loading in zeolites was measured by inductively coupled plasma atomic emission spectroscopy with an ICP-OES SPECTRO BLUE TI spectrometer (Kleve, Germany). The gold phase was extracted from the sample by means of aqua regia.

The amount of nitrogen in the samples was examined by elemental analysis (Elemental Analyser Vario EL III, Elementar Corporation, Langenselbold, Germany).

^11^B MAS NMR studies were performed on a Bruker NMR 500 MHz spectrometer (11.4 T) (Billerica, MA, USA) at a resonance frequency of 160.46 MHz using a pulse of 0.4 µs (π/11). The chemical shift was determined using boric acid as a reference. All samples were hydrated prior to measurement by placing them overnight in a desiccator with a saturated solution of magnesium nitrate.

Infrared spectra combined with pyridine adsorption were recorded using a Bruker Invenio FTIR spectrometer (Billerica, MA, USA) with an in situ vacuum cell (resolution 4 cm^−1^, number of scans = 64). Solids in the form of thin wafers (ca. 10 mg cm^−2^.) were placed inside the cell and then evacuated at 350 °C for 2 h. After that, pyridine was admitted at 150 °C. After saturation with pyridine, the solids were degassed at 150, 200, 250 and 300 °C in vacuum for 30 min at each temperature. The spectrum without adsorbed pyridine (after sample activation) was subtracted from all recorded spectra. The numbers of Lewis and Brønsted acidic sites were calculated assuming the extinction coefficient ε for the band at ca 1545 cm^−1^ = 0.044 cm^2^ μmol^−1^ (Brønsted acidic sites) and 1450 cm^−1^ = 0.165 cm^2^ μmol^−1^ (Lewis acid sites) [23].

The attenuated total reflectance–Fourier transform infrared (ATR–FTIR) spectra measurements were performed on a Bruker Vertex 70 spectrometer (Billerica, MA, USA) equipped with an ATR attachment with a diamond crystal plate. The catalysts in portions of 15 mg were treated with 0.5 mL of 0.2 M glucose solution and heated at 80 °C, then placed over the diamond crystal, and force was applied to the sample by rotation of the pressure clamp to its click-stop release. The spectra were recorded in the range from 4000 to 400 cm^−1^ (resolution 4 cm^−1^, number of scans = 64). A background air spectrum was subtracted from each spectrum of the solid sample.

### 2.7. Glucose Oxidation

Glucose oxidation reactions with molecular oxygen as an oxidant were carried out in a pressure batch reactor from Parr (Moline, IL, USA), equipped with temperature and pressure controllers. In a typical experiment, 20 mL of 0.2 M aqueous glucose solution and the corresponding zeolite catalyst in the amount to achieve the 1970/1 glucose/Au molar ratio, were added to the reactor. The reaction was performed without base addition. After closing, the reactor was purged several times with oxygen before reaction, then it was heated up to 110 °C (a heating rate 2 °C min^−1^) and pressured at 0.5 MPa with molecular oxygen. The reactions were conducted for 60 min. After the reaction, oxygen was released and the reactor was cooled down to room temperature. Afterwards, the reaction mixtures were withdrawn using a syringe, filtered through a Millipore filter (0.2 μm, PTFE, Maidstone, Great Britain) and analyzed by ultrahigh performance liquid chromatography on a UPLC Acquity Arc Waters instrument (Milford, MA, USA).

To investigate time-dependent glucose conversion, the reactions were performed over selected zeolite catalysts (Au/MCM-36 and Au/NH_2_/MCM-36) for 15, 30, 60, 120 and 180 min (reaction conditions: 20 mL of 0.2 M glucose solution, glucose/Au (molar ratio) = 1970/1, T = 110 °C, p O_2_: 0.5 MPa, stirring rate: 600 rpm). The kinetics of glucose oxidation over the selected catalysts was estimated on the basis of pseudo-first order model expressed by the following equation: $ln\left(C/C_{0}\right)=–kt$ where $C_{0}$ is the initial concentration of glucose, C is the glucose concentration after given reaction time, k is the reaction rate constant and t is the time of the reaction. According to this model, the reaction rate constant can be determined by drawing a plot of $-ln\left(C/C_{0}\right)$ versus t, whose slope is equal to the value of k. R^2^ coefficients were determined to assess the goodness of fit to the pseudo-first order model.

Microwave-assisted oxidation of glucose with hydrogen peroxide as an oxidant. The reaction tests were performed in a microwave synthesis platform MicroSYNTH (from Milestone, Sorisole, Italy) equipped with a contactless temperature infrared controller. Glucose aqueous solution in a portion of 7.64 mL (0.3243 g of glucose in deionized water) was introduced into a 10 mL glass tube. Then, an appropriate amount of a given catalyst (Au/glucose molar ratio: 1970/1) was added. Finally, 0.36 mL of a hydrogen peroxide solution was introduced. After the oxidant introduction, the glucose concentration was of 0.2 M, and the H_2_O_2_ to glucose molar ratio was equal to 2.2. The reactions were conducted for 10 min at 110 °C (stirring rate: 80% of the maximum setting). The post-reaction mixture processing and analysis were the same as in the case of glucose oxidation with the use of molecular oxygen.

### 2.8. Analysis of Reactant and Products

The quantitative analyses of the reaction mixtures were performed using an ultrahigh performance liquid chromatograph UPLC Acquity Arc Waters (Milford, MA, USA) and the products of the reaction were analyzed by two detectors, refractive index (RI 2414) and a photodiode array (PDA 2998). The reactant and the products were separated on a Shodex sugar column SH1011, heated at 30 °C. The eluent was an aqueous solution of H_2_SO_4_ (0.005 M) and its flow rate was set at 0.6 mL min^−1^. The samples to be analyzed were collected at the end of the reaction: 1 mL of the reactant/products solution was diluted in 50 mL of deionized water.

## 3. Results and Discussion

### 3.1. Composition of Catalysts and Their Textural/Structural Properties

**Figure 1:**
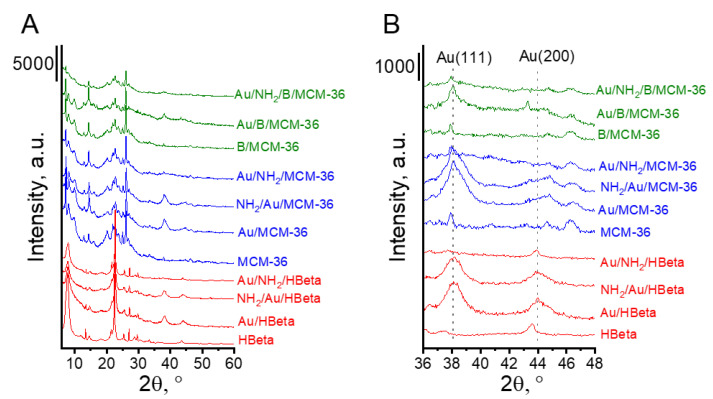
XRD patterns of investigated catalysts. (**A**) The range of 6–60° 2*θ*; (**B**) the range of XRD peaks typical of Au(111) and Au(200) planes.

The textural and structural parameters of the prepared catalysts were characterized by XRD and low temperature adsorption/desorption of nitrogen. Figure 1 presents the diffractograms of MCM-36 and HBeta zeolites before and after modifications with boron, amino-organosilane species and gold. All zeolites showed well-defined crystal structures. The XRD patterns present reflections typical of MCM-36 and HBeta zeolite structures. It is worth noting that additional reflections characteristic of metallic gold can be observed in the XRD patterns of the calcined gold catalysts. Modification of MCM-36 zeolite with boron did not change the zeolite structure. The XRD pattern of B/MCM-36 (Figure 1) did not exhibit the presence of boron oxide or boron acid crystal phases. To examine in detail the localization of boron species in MCM-36 zeolites, the ^11^B MAS NMR studies were performed. The ^11^B MAS NMR spectra of boron containing MCM-36 (see Supplementary Data; Figure S1) showed two signals in the region characteristic of boron species in tetrahedral environments. The most intensive signal centered at around 2 ppm according to literature [24,25] is assigned to the framework-related tetrahedral deformed zeolite [HO-B-(OSi)_3_]^−^. This result indicates that the applied modification method led to partial replacement of aluminum in the zeolite skeleton with boron.

Summarizing the modification with boron, amine species and gold do not adversely affect the structural properties of the zeolites but have a significant influence on the textural properties. In Table 1, the texture parameters of all prepared materials are collected. The addition of gold or especially amino-organosilane (APMS) to each type of support (HBeta, MCM-36, B/MCM-36) caused a decrease in the BET surface area, external surface area and pore volume. Significant decrease in the textural parameters of zeolites after APMS modification indicates that the amine species partially block the zeolite pores. This effect was the most pronounced for NH_2_/Au/Beta in which the zeolite was modified with amino-organosilane after gold loading and calcination.

Catalyst composition estimated from ICP-OES for gold and elemental analyzes for nitrogen is shown in Table 1. The catalysts based on aluminosilicate HBeta and MCM-36 zeolites have gold content in the range of 1.78–2.37 wt.%. The boron containing zeolite showed a lower efficiency of gold introduction (1.55 and 1.58 wt.%). Moreover, the amount of gold is similar for the non-calcined gold zeolite containing amino-organosilane (Au/NH_2_/B/MCM-36) and calcined Au/B/MCM-36. A different tendency was observed for the gold catalyst based on aluminosilicate zeolites (HBeta, MCM-36). The applied method of modification with amino-organosilane affects the final content of gold in the catalysts. The highest content of gold was observed in the non-calcined gold zeolites containing amino-organosilane. The results of elemental analysis indicate insignificant difference in nitrogen content in the non-calcined gold zeolites containing amino-organosilane (Au/NH_2_/zeolite) and the calcined gold zeolites modified with amino-organosilane after gold loading (NH_2_/Au/zeolite), based on the same type of zeolites.

### 3.2. Surface Properties of Catalysts

#### 3.2.1. Acidity of Selected Catalysts

The acidic properties of zeolite supports and calcined gold zeolites were characterized by FTIR spectroscopy with pyridine adsorption (Figure S2). The studies were not performed for the zeolites containing amino-organosilane due to a relatively low decomposition temperature of the modifier. It is known that pyridine is a suitable probe molecule to detect the presence of both Brønsted (the bands at 1545 and 1620 cm^−1^ from protonated pyridine) and Lewis (the bands at 1455 and 1610 cm^−1^ from coordinative bonded pyridine) acidity [26,27,28]. In the spectra of all zeolites (before and after gold introduction) after pyridine adsorption and evacuation at 200, 250 and 300 °C (it means when the bands at 1447 and 1597 cm^−1^ from pyridine hydrogen bonded to silanol groups disappeared), the bands from Brønsted acid sites (BAS) and Lewis acid sites (LAS) are well visible (Figure S2). However, these bands are more intense for the supports than for the gold-containing zeolites. The number of BAS for all zeolites studied was estimated on the basis of pyridine chemisorbed after evacuation at 150, 200, 250 and 300 °C, whereas the number of LAS–after evacuation at 300 °C (Table 2). Moreover, the ratio of pyridine chemisorbed on BAS/LAS is shown. As follows, the BAS/LAS ratio is much higher for all zeolites of MCM-36 type (between ca 4–6) than for the HBeta samples (ca. 1.5) and it is higher for the supports than for gold-zeolites. It indicates that the number of BAS is higher on MCM-36 than on HBeta samples in relation to the number of LAS and that the number of BAS decreased after modification of both supports with gold. Interestingly, the introduction of boron into the framework of MCM-36 does not significantly change the BAS/LAS ratio (5.73 for MCM-36 and 6.31 for B/MCM-36), the numbers of BAS and LAS in both zeolites are comparable. Moreover, the gold loading on MCM-36 and B/MCM-36 only slightly decreased the number of BAS and therefore, their numbers are almost the same on Au/MCM-36 and Au/B/MCM-36 (3.77 and 4.07, respectively). The strength of BAS was estimated as the percentage of the amount of pyridine desorbed from BAS after evacuation at 300 °C relative to that desorbed at 200 °C. The higher the percentage of pyridine desorbed, the lower the strength of BAS. The data shown in Table 2 indicate that the strength of BAS is very high and comparable for MCM-36 and Au/MCM-36 (22.4 and 24.7% of desorbed pyridine, respectively). The modification of MCM-36 with boron significantly increased the strength of BAS on B/MCM-36 (5.3% of desorbed pyridine), but the modification of this support with gold again led to a decrease in the BAS strength (35.2% of desorbed pyridine for Au/B/MCM-36). The Brønsted acid sites present on HBeta and Au/HBeta are characterized by much lower strength than those on the corresponding MCM-36 and Au/MCM-36 zeolites.

#### 3.2.2. Particle Size and Oxidation State of Gold

The oxidation state of gold in zeolites was determined on the basis of complementary measurements of XRD patterns, UV–vis spectra, HR-TEM images and XPS study. Moreover, the particle size of gold was measured on the basis of HR-TEM images.

The presence of metallic gold in the samples studied was confirmed by all techniques used. The diffraction peaks at 2θ = 8.2° and 44.4°, typical of metallic gold [29], were found in the XRD patterns of gold-containing zeolites after calcination (Au/HBeta, Au/MCM-36, Au/B/MCM-36) and the calcined samples further modified with the amino-organosilane (NH_2_/Au/HBeta and NH_2_/Au/MCM-36) (Figure 1B). In the XRD patterns of gold non-calcined zeolites functionalized with APMS and modified with gold (Au/NH_2_/HBeta, Au/NH_2_/MCM-36, Au/NH_2_/B/MCM-36) the reflections corresponding to metallic gold were not detected, suggesting the presence of very small gold crystallites.

**Figure 2:**
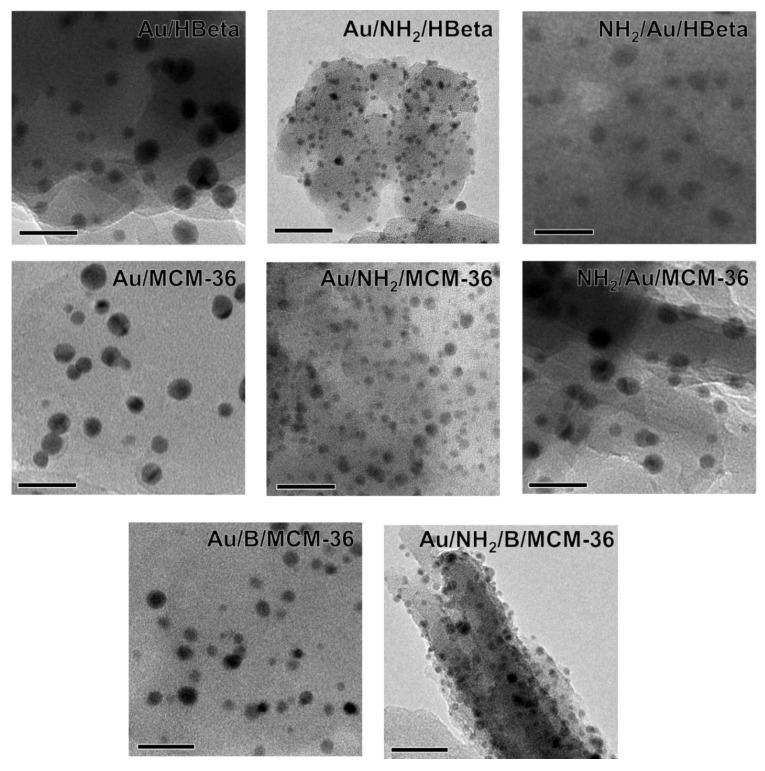
HR-TEM images of investigated gold catalysts. Scale bars are equal to 20 nm in each case.

**Figure 3:**
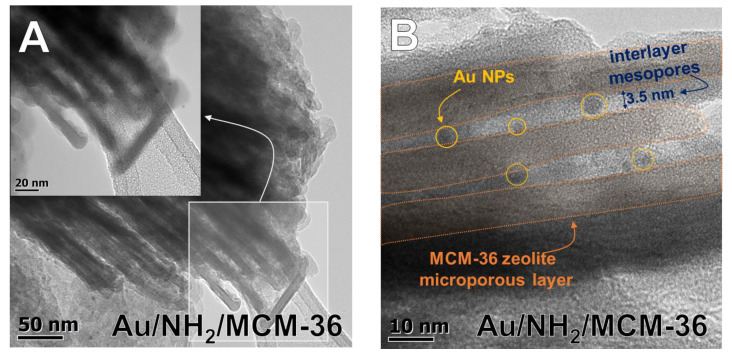
(**A**) An exemplary HR-TEM image indicating layered structure of MCM-36 support; (**B**) HR-TEM image presenting Au NPs location in interlayer spaces of MCM-36 support.

**Figure 4:**
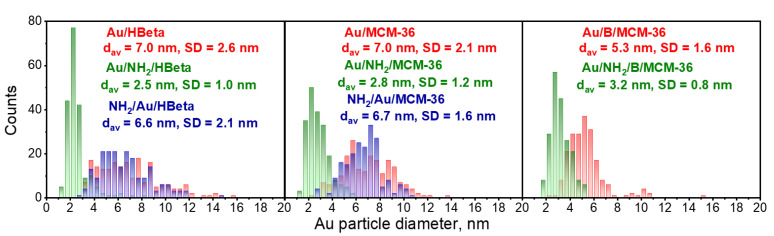
Gold particle size distribution histograms of the catalysts. The values of mean Au NPs diameter (d_av_) and standard deviation (SD) were included. The number of particles measured was 200 in each case.

To confirm the above suggestions, HR-TEM images were taken. They allowed us to get the information about the shape of gold crystallites, average gold particle size and distribution of Au NPs on the surface of zeolites, depending on the preparation conditions. Spherical metallic gold nanoparticles (NPs) were well visible in the HR-TEM images of all gold catalysts (Figure 2). In the MCM-36 structure, gold was localized in mesopores between zeolites layers (Figure 3). The average size of gold particles is shown in Table 1 and the Au NPs distributions histograms, plotted on the basis of HR-TEM images, are shown in Figure 4. The influence of calcination process after functionalization of zeolite with APMS and gold introduction is clearly seen. Gold particles on the surface of calcined catalysts (Au/HBeta, Au/MCM-36, Au/B/MCM-36) were much larger (d_av_ = 5–7 nm) than the particles on non-calcined gold-catalysts containing APMS (Au/NH_2_/HBeta, Au/NH_2_/MCM-36, Au/NH_2_/B/MCM-36) (d_av_ = 2.5–3.5 nm) indicating that Au agglomeration took place during calcination. The difference between both kinds of samples is well visible in histograms. They show a wide Au NPs size distribution for the calcined samples (the particles diameter oscillates between 3 and 12 nm) and clearly narrower particle size distribution for the non-calcined zeolites prepared without calcination (between 1–6 nm). The results obtained are consistent with the literature data [30] which demonstrated a similar narrow particle size distribution for Pd NPs loaded on silica functionalized with APMS. The treatment of calcined samples with APMS did not change significantly the size of gold particles.

**Figure 5:**
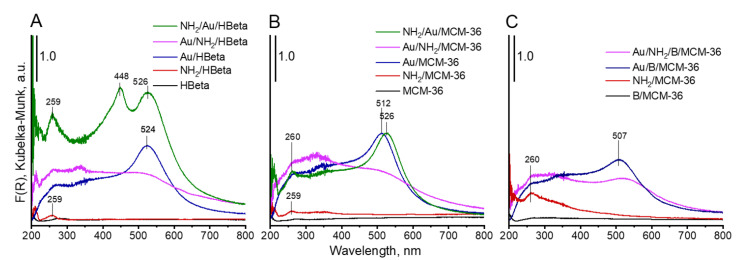
DR-UV-vis spectra of prepared materials.

UV-vis spectroscopy was used as the next complementary technique for gold characterization. The UV-vis spectra of all Au-containing samples show the characteristic surface plasmon resonance (SPR) band at ca 500 nm, typical of metallic gold [31] (Figure 5). However, the differences in intensity of the SPR band are observed, depending on gold-zeolite composition and preparation procedure. It is known from literature [32,33] that the intensity of SPR band is related to the diameter of gold nanoparticles. The intensity of the band increases with increasing size of metal particles. The small intensity of the band at around 512–520 nm, observed in the spectra of non-calcined catalysts in comparison to that of the analogous band in the spectra of calcined samples, indicates the presence of very small gold crystallites in Au/NH_2_/HBeta and Au/NH_2_/MCM-36. Similar intensities of the band at around 520 nm in the UV-vis spectra of calcined gold-zeolites and that in the spectra of calcined gold-zeolites further treated with APMS, indicate that the introduction of amino-organosilane after sample calcination did not influence the size of metal particles. These results are in line with the results of XRD study and TEM measurements. However, it is important to note that the presence of organosilane (APMS) in zeolites leads to the appearance of new bands in the UV-vis spectra. Two bands at ca 200 and 260 nm are characteristic of amine species [34,35,36]. Moreover, an additional band at 448 nm is visible in the spectrum of NH_2_/Au/HBeta. The appearance of a secondary peak in the UV-vis spectrum at a shorter wavelength relative to the typical gold SPR band position was observed earlier for gold core–mesoporous silica shell nanoparticles (Au-MMS-rods) [37]. On the other hand, it has been documented that gold nanospheres show one absorption peak in the UV-vis spectrum, while nanorods show two characteristic absorption peaks due to transverse plasmon resonance and longitudinal plasmon resonance [37]. That is why, in the case of zeolites with spherical particles, the presence of the secondary band cannot be related to transverse/longitudinal SPR and should be a result of amino-organosilane localization in the vicinity of gold. The SPR band is sensitive to dielectric properties of the environment in the vicinity of the illuminated nanoparticle. Changes in the environment of the nanoparticle cause a shift in the maximum of the plasmon absorption band. Taking into account that the secondary band in the shorter wavelength region is not present in the spectrum of NH_2_/Au/MCM-36, one can suggest the presence of gold particles in two different surroundings only on the surface of NH_2_/Au/HBeta. Most probably on the surface of NH_2_/Au/HBeta zeolite, APMS anchors to the hydroxyls which are close to gold and that is why amino-organosilanes are in very close proximity to gold changing dielectric properties of gold environment (the SPR band at 448 nm). In the layered and pillared MCM-36, gold is preferentially localized in mesopores (between zeolite layers) and APMS is anchored to hydroxyl groups relatively away from the gold. As a result, only one Au SPR band at ca 520 nm is visible in the spectra of NH_2_/Au/MCM-36.

**Figure 6:**
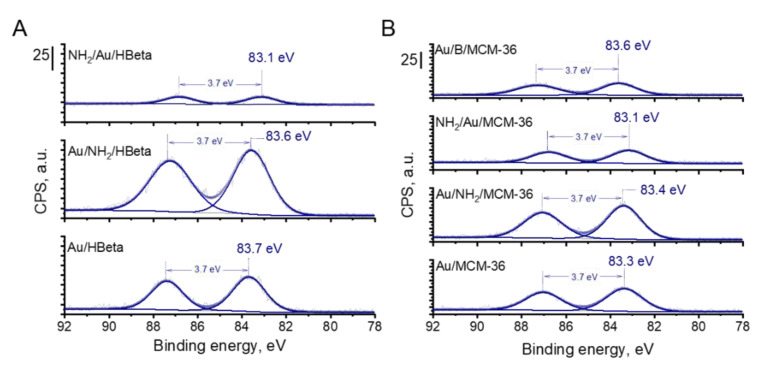
Au 4f region of XP spectra of gold-containing zeolites from (**A**) HBeta; (**B**) MCM-36 and B/MCM-36 series. The values of binding energy corresponding to the Au 4f_7/2_ peaks are marked.

The presence of metallic gold particles on the surface of all catalysts was further confirmed by X-ray photoelectron spectroscopy. The XPS spectra of gold zeolites based on HBeta and MCM-36 are compared in Figure 6. In all Au 4f spectra, two components separated by 3.7 eV, namely Au 4f_7/2_ and Au 4f_5/2_, were identified. According to literature [38], the Au 4f_7/2_ peak of metallic gold species corresponds to the binding energy value of 84.0 eV. Interestingly, for all materials prepared in this study, the binding energy corresponding to the Au 4f_7/2_ peak was lower than 84.0 eV, indicating that in all the samples the gold species were present in metallic form on whose surface negative charge was accumulated [39]. The higher the BE shift towards lower binding energy value of Au 4f_7/2_, the higher the negative charge accumulated on the surface of metallic gold species [39]. From among all calcined gold catalysts, the lowest BE of Au NPs was observed for Au/MCM-36 catalyst (BE = 83.3 eV; Figure 6). For Au/B/MCM-36 and Au/HBeta the BE of Au NPs was slightly higher and took the values of 83.6 and 83.7 eV, respectively. As far as the electronic properties of gold species are concerned, it is important to underline that Au NPs in the non-calcined gold catalysts were characterized by similar binding energy values as those determined for the calcined samples. Thus, in view of this observation, one can conclude that the presence of amine group has no significant impact on the electronic properties of gold species. Interestingly, in the calcined gold catalysts modified with amino-organosilane, the binding energy of gold species was the lowest from among all materials synthesized in this work, suggesting the presence of strong electronic interaction between the amino-organosilane and Au NPs. Thus, these results show the difference between the amine species on gold zeolites calcined prior to modification with APMS and those on the non-calcined (containing amino-organosilane) samples.

### 3.3. Glucose Oxidation with Molecular Oxygen

#### 3.3.1. Influence of Zeolite Support on the Activity of Gold Catalysts

Table 3 shows the results of glucose oxidation after 1 h of the reaction performed over all catalysts studied in this work. Independently of the glucose conversion, the selectivity to gluconic acid over gold catalysts was very high (ca. 99%). All zeolite supports, i.e., HBeta, MCM-36 and B/MCM-36, exhibited low glucose conversion. Deposition of gold species on the zeolite supports grafted with APMS and subjected to calcination (the gold calcined zeolites) resulted in a significant increase in the catalysts activities. In view of these results, one can conclude that zeolite supports were almost inactive in glucose oxidation and the main active components responsible for efficient glucose oxidation to gluconic acid were gold nanoparticles. From among all calcined gold zeolites, the highest glucose conversion of 65.2% was observed for Au/MCM-36 (Table 3, Entry 6). Interestingly, in the case of Au/HBeta, which had the same average gold particle size as that of Au/MCM-36, glucose conversion was significantly lower and was found to be of 50.6% (Table 3, Entry 2). In view of these results, one can conclude that the type of zeolite support had a significant impact on the activity of gold particles. The gold catalysts supported on MCM-36, characterized by larger pore size and stronger Brønsted acidity (see Table 1 and Table 2), were found to be more active than those supported on HBeta. It shows that the porosity of the support and the strength of its BAS can have significant impact on the activity of gold nanoparticles in glucose oxidation. Interestingly, the lowest glucose conversion of 45.8% was observed for Au/B/MCM-36. This catalyst had slightly smaller gold particle size than Au/MCM-36 (5.3 vs. 7.0 nm, respectively), the same pore structure, almost the same number of BAS but a weaker Bronsted acidity (see Table 2). Since smaller gold particles should result, in general, in higher glucose conversion than that observed for the catalysts with larger gold particles [15], one can expect that a lower glucose conversion in the presence of Au/B/MCM-36 containing smaller Au NPs can be assigned to the lower strength of acid sites. According to previous reports [17,40,41], BAS can play an important role in transformation of glucose molecule to geminal 1,1-diol (hydrate), which is then oxidized by active oxygen species adsorbed on gold particles. The stronger the BAS, the more labile the protons, and thus, the more efficient the protonation of glucose molecule to form *gem*-diol. As far as differences in the activity of calcined gold zeolites are concerned, it is important to underline that Au/MCM-36, Au/B/MCM-36 and Au/HBeta exhibited different electronic properties of gold species. As described in the XPS section, Au NPs supported on Au/MCM-36 sample were characterized by significantly lower binding energy than that of the gold species in Au/B/MCM-36 and Au/HBeta (83.3 vs. 83.6 and 83.7 eV, respectively; Figure 6). According to literature [39], a shift of BE of gold species towards binding energy values lower than 84.0 eV may indicate accumulation of partial negative charge on the surface of metallic gold species. The stronger the BE shift, the stronger the partial negative charge accumulated on Au NPs. Formation of metallic gold species with partial negative charge on their surface has been previously reported by Xu et al. [39]. These authors have established that accumulation of negative charge on the surface of Au NPs facilitated activation of molecular oxygen and increased the activity of gold catalysts in oxidation reactions. Thus, in view of previous literature reports, we hypothesize that the highest activity of Au/MCM-36 may result not only from different porosity and acidity of the supports in comparison to Au/HBeta, but also from different electronic properties of the active phase, i.e., the gold species. Since Au/B/MCM-36 and Au/MCM-36 exhibited the same pore structure, but different electronic properties of gold species, one can expect that this hypothesis is very probable. The role of partial negative charge on the surface of gold species in oxidation of glucose will be discussed below.

As described in Section 3.2.2., non-calcined gold catalysts contained significantly smaller gold particles than the calcined catalysts. Thus, one can expect that smaller gold particle size should result in a higher catalytic activity. Indeed, it was revealed that the activity of all non-calcined gold catalysts after 1 h of the reaction was much higher than that observed for calcined samples. From among all non-calcined samples, the highest glucose conversion of 77.5% was observed for Au/NH_2_/MCM-36 catalyst. It is worth noting that amine-grafted zeolite supports exhibited almost the same activity as parent zeolites (results not shown). Thus, the amine groups themselves were not able to take part in oxidation of glucose to gluconic acid. The possible synergistic interaction between amine groups and Au NPs, which could influence the gold activity, has been excluded. As described in the XPS section, the presence of amine group of APMS in the neighborhood of gold species has no impact on the electronic properties of gold species which were similar in Au/MCM-36 and Au/NH_2_/MCM-36.

To shed more light on the role of amine groups in glucose oxidation over gold catalysts, additional samples, in which APMS was grafted on the surface of calcined samples, were synthesized (NH_2_/Au/HBeta and NH_2_/Au/MCM-36) and had similar average gold particle size as the parent samples before anchoring of APMS (see Table 1 and Figure 4). As implied by Table 3 data, the influence of APMS grafting on the activity of calcined gold catalysts was strongly affected by the type of zeolite support. In the case of the catalysts supported on HBeta, modification with APMS significantly diminished the catalytic activity of the parent sample. Interestingly, in the case of NH_2_/Au/MCM-36, modification with APMS had negligible influence on glucose conversion. In view of these results, one can conclude that the type of zeolite support had a significant impact on the role of amine groups, and thus, on the overall catalytic performance of the catalysts. This hypothesis was further confirmed by different optical properties of gold species in NH_2_/Au/HBeta and NH_2_/Au/MCM-36 (see Figure 5). As described in the TEM section, the most important difference between Au-HBeta and Au-MCM-36 was localization of gold species. In Au/HBeta, gold catalysts were localized mainly on the external surface of the support (see Figure S3), while in Au/MCM-36 they were localized mainly between the zeolite layers (see Figure 3B). Taking into account the results of UV-vis measurements, it is very likely that gold particles in NH_2_/Au/HBeta were covered by APMS, while in NH_2_/Au/MCM-36 the distance between the Au NPs localized inside the zeolite layers and APMS modifier was much larger, preventing deactivation of the gold species. Since glucose conversion in the presence of NH_2_/Au/MCM-36 was only slightly lower than that in the presence of Au/MCM-36, we inferred that deactivation of NH_2_/Au/HBeta resulted from covering of gold particles with the amino-organosilane, but not from the strong interaction between the amine group of APMS and the gold species. Moreover, a higher activity of non-calcined gold catalysts (Au/NH_2_/zeolite) than that of calcined NH_2_/Au/zeolite materials indicated that the role of amino-organosilane in both types of materials (the calcined catalysts modified with amino-organosilane and non-calcined ones) was different.

Differences in activities of non-calcined gold zeolites containing amino-organosilane (Au/NH_2_/MCM-36 and Au/NH_2_/HBeta) and calcined gold zeolites modified with amino-organosilane after gold loading (NH_2_/Au/MCM-36 and NH_2_/Au/HBeta) can be explained on the basis of ATR spectra of zeolites after adsorption of glucose solution and drying at 80 °C. As can be seen in Figure S4, the FTIR spectra of the zeolites and glucose show the main bands in the wavenumber range 1300–700 cm^−1^. The characteristic bands assigned to glucose have maxima at 1222 and 1200 (δCH + δOH in plane), 1148, 1050 and 994 (νCO + νCC), 1106 and 1021 (νCO), 915 (νCO + νCCH + γ_as_ ring of pyranose ), 838 (δOH) and 772 cm^−1^ (δCCO + δCCH) [42]. Some of these bands (1200, 1148, 1106, 1021, 994, 915 and 772 cm^−1^) are also visible in the spectra of the zeolites treated with glucose, but they are shifted to the lower wavenumbers (1190, 1145, 1077, 1017, 985, 900 and 770 cm^−1^, respectively). The highest blue-shift is observed for the bands that are overlapped by those assigned to the T-O-T (T = Si or Al) vibrations in the zeolites (at 1220, 1087 and 797 cm^−1^). The latter are also shifted in the spectra of the zeolites after glucose adsorption (to ca 1212, 1048 and 793 cm^−1^, respectively). It is important to note that the spectra of all zeolites studied after glucose adsorption are similar in the range 1300-700 cm^−1^.

Significant differences in the ATR spectra are observed in the C=O vibration region (1800–1600 cm^−1^). Two bands at ca 1737 and 1777 cm^−1^ are present only after adsorption of glucose on Au/NH_2_/MCM-36 and Au/NH_2_/HBeta. The same bands were previously observed in the spectra of gold-zeolites after glucose oxidation and were assigned to C=O stretching vibrations in the reaction product—gluconic acid [17]. In the ATR study in this work, the oxidation of glucose cannot be considered, but the above-mentioned bands can be assigned to chemisorption of glucose. Such a chemisorption with the participation of protons and water had been proposed earlier [17] and the coordination of glucose-water-proton complex to the zeolite surface had been shown. The glucose-water-proton complex contains carboxyl groups whose C=O vibrations appear in the IR spectrum as the two, above-mentioned bands. The question is why such a chemisorption was observed only on two catalysts. The chemisorption of this type cannot be explained only as due to the presence of amine groups on the zeolite surface as the two bands were not observed in the spectra of NH_2_/Au/zeolite samples. Therefore, we hypothesize that the amine groups in Au/NH_2_/zeolite are protonated during the procedure of gold anchoring (from auric acid precursor). It is known that in the process of gold anchoring on APMS functionalized samples in the first step amine groups are protonated and AuCl_4_^−^ anion is connected to the NH_3_^+^ moiety [43,44]. The hypothesis about partial protonation of the amine groups of APMS in non-calcined gold catalysts was confirmed by XPS. According to literature [45], the amine groups of APMS on functionalized surfaces are characterized by the binding energy of ca 399.1–399.6 eV, while the protonated amine groups are characterized by the binding energy of ca 400.9–401.7 eV. As can be seen from Figure S5, the N 1s region of the non-calcined gold catalysts and the calcined gold catalysts modified with amino-organosilane differed substantially. For the non-calcined samples, one can observe a more pronounced peak with the binding energy typical of protonated amine groups (region highlighted in yellow). Interestingly, the highest relative contribution of protonated amine groups was observed for Au/NH_2_/HBeta catalyst, indicating different efficiency of amine protonation in MCM-36 and HBeta catalysts. In view of these results, we hypothesize that in the non-calcined samples (Au/NH_2_/zeolite) the protonated amine groups take part in the chemisorption of glucose via aldehyde groups. It is not the case for the catalysts in which APMS was added after gold loading followed by calcination.

Taking into account the above described results one can conclude that the enhancement in glucose conversion in the presence of Au/NH_2_/zeolite is caused, to some extent, by the increase in the glucose chemisorption rate attributed to the presence of protonated amine groups.

Normalization of catalysts activity in glucose oxidation to the exposed surface gold atoms expressed in turnover frequency, TOF, provides more detailed insight into the reactivity of Au NPs on the surface of the catalysts (see Table 3). It was found that the highest reactivity of surface gold atoms was observed for calcined gold catalysts characterized by gold particle size in the range from 5.3 to 7.0 nm. It is important to emphasize that for all calcined samples, TOF values are strongly correlated with electronic properties of gold species. The lower the BE of the gold species, the higher the TOF values. These results clearly show that accumulation of partial negative charge on the surface of metallic gold species is one of the most important factors determining the activity of surface gold atoms in glucose oxidation. For non-calcined samples, in which gold particles were much smaller (ca. 2.5–3.2 nm in diameter; Table 1), TOF values were significantly lower. Thus, the results obtained in this study are in agreement with the previous literature reports [17], in which the highest reactivity of surface gold atoms was observed for gold particles with diameter of ca 30 nm, and the TOF value was reduced as the gold particle size decreased.

In order to evaluate the catalytic activity of the Au/MCM-36 (i.e., the catalyst with the highest TOF value) in base-free glucose oxidation with molecular oxygen, several recent reports on this topic were analyzed. Table 4 contains a comparison of selected parameters characterizing gold catalysts (on various supports such as zeolites (MCM-36 and HBeta) [17], titania (TH) [4], mesoporous carbon (CMK) [46], mesoporous silica (SBA-15) [47], hydroxyapatite-layered double hydroxide composite (HAP-LDH) [48]) and their catalytic performance in base-free glucose oxidation. The most active catalyst from each work was selected for this comparison. It is noteworthy that Au/MCM-36 is characterized by the highest TOF value from all the corresponding catalysts, even though it contains the largest Au NPs.

#### 3.3.2. Time Dependence of Glucose Conversion

**Figure 7:**
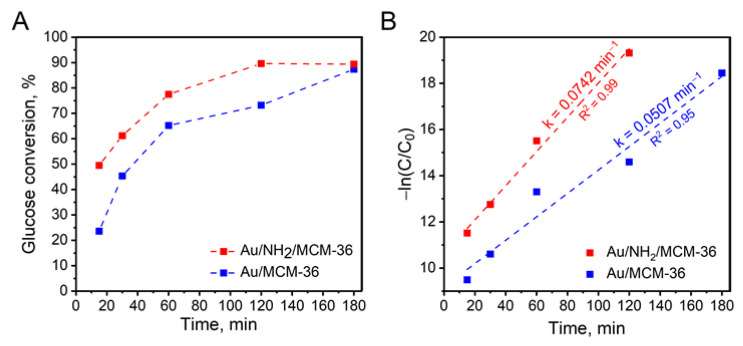
(**A**) Effect of the reaction time on the activity of Au/MCM-36 and Au/NH_2_/MCM-36 in base-free glucose oxidation with molecular oxygen; (**B**) pseudo-first order plot for determination of glucose oxidation rate. The reaction rate constants (*k*) were marked in the graph. *Reaction conditions:* 20 mL of 0.2 M glucose solution, glucose/Au (molar ratio) = 1970/1, mixing rate = 600 rpm, T = 110 °C, p O_2_ = 0.5 MPa, time = 1 h; A—gluconic acid, B—glucuronic acid.

To get a deeper insight into glucose oxidation over the most active gold catalysts supported on MCM-36 zeolite, kinetic measurements were performed. Figure 7A shows glucose conversion over Au/MCM-36 and Au/NH_2_/MCM-36 samples as a function of reaction time. It was found that the non-calcined catalyst, irrespectively of the reaction time, exhibited significantly higher glucose conversion than that observed for the calcined one. As can be seen from Figure 7A, Au/NH_2_/MCM-36 reached the reaction equilibrium after 120 min, while the same glucose conversion over Au/MCM-36 was observed 60 min later, i.e., after 180 min of the reaction. To shed more light onto the kinetics of glucose oxidation over gold catalysts supported on MCM-36, the experimental data were fitted to pseudo-first order kinetic model (see Figure 7B). It was found that the experimental data obtained for the catalysts fit in within this model. For the reactions with the use of Au/MCM-36 and Au/NH_2_/MCM-36, the R^2^ values were higher than 0.95 (see Figure 7B). The highest reaction rate constant of 0.0742 min^−1^ was observed for Au/NH_2_/MCM-36 sample, and was ca 1.5 times higher than that estimated for Au/MCM-36.

### 3.4. Glucose Oxidation with Hydrogen Peroxide

Table 5 summarizes the results of the microwave-assisted oxidation of glucose with the use of hydrogen peroxide as an oxidant. The glucose conversion in the presence of zeolite supports was relatively low and did not exceed 15%. Only gold catalysts showed significant activities. The selectivity to gluconic acid for the gold-containing zeolites was high and reached >98% in each case.

The dependence of glucose conversion on the structural/textural properties of calcined gold zeolites in the microwave-assisted glucose oxidation with the use of hydrogen peroxide appeared to be similar to that reported above for glucose oxidation with the use of molecular oxygen. The activities of the Au/HBeta and Au/MCM-36 gold zeolites differed even though their gold particle size was the same (ca. 7 nm). The glucose conversion higher by ca 10 percentage points was observed for Au/MCM-36 catalyst. This difference can be attributed to the larger pore size in the latter sample. However, the impact of electronic properties of Au NPs and acidic centers on the zeolite supports on the activity in glucose oxidation with H_2_O_2_ should also be taken into account. According to the literature data [49], hydrogen peroxide is chemisorbed on Au surface active species. The chemisorption is accompanied by decomposition of H_2_O_2_. One of the proposed mechanisms of chemisorption is the redox mechanism [49] in which electron transfer from gold species to H_2_O_2_ occurs, which leads to the formation of OH^−^ ions and HO^•^ radicals. The OH^−^ ions react with free H_2_O_2_ to yield HO_2_^−^ and H_2_O. Finally, in the reaction between HO_2_^−^ and HO^•^ radicals, O_2_ and H_2_O are generated. The higher the electron density on the surface of Au NPs, the faster the electron transfer in the first step of this redox pathway. As the electron density (concluded from XPS results) on the calcined and non-calcined gold zeolites based on HBeta was lower than that on the catalysts based on MCM-36 and the catalysts of the latter series were more active, one can conclude that the rate of hydrogen peroxide decomposition is crucial for the activity of gold zeolites in glucose oxidation with H_2_O_2_. The acidity of the support for gold seems not to play an important role in this reaction because Au/HBeta with a higher concentration of BAS showed a lower activity than Au/MCM-36 containing a lower number of BAS. Moreover, the presence of protonated amine groups grafted on the zeolitic surface in the non-calcined gold catalysts did not result in reaching distinctly superior glucose conversion when compared to that of the calcined ones, as reported for the oxidation with molecular oxygen. A slight activity enhancement was observed only for Au/NH_2_/B/MCM-36 when compared that of Au/B/MCM-36 (56.6% vs. 49.5%, respectively). It suggests that the rate limiting reaction step in the oxidation with H_2_O_2_ is different from that in the oxidation with molecular oxygen.

Modification of the calcined Au/zeolites by APMS led to different activities depending on the zeolite structure. As mentioned before, it is expected that in NH_2_/Au/HBeta sample, Au NPs are covered with the amine modifier (as concluded from UV-vis results) and therefore, the activity significantly decreased in both the oxidation with H_2_O_2_ as well as with O_2_. It is not the case for NH_2_/Au/MCM-36 and, interestingly, a decrease in NH_2_/Au/MCM-36 activity after the amino-organosilane modifier introduction is relatively low.

Similarly to the above-mentioned results for glucose oxidation with O_2_, the highest TOF values (Table 5) were observed for the calcined gold zeolites containing the largest Au NPs, i.e., those of ca 7 nm in diameter (Au/MCM-36 and Au/HBeta). Slightly lower TOF number were obtained for the amino-organosilane-grafted NH_2_/Au/MCM-36 catalysts. In contrast, the lowest activity expressed by the TOF number was found to show NH_2_/Au/HBeta.

The glucose conversions for all the examined gold catalysts did not exceed 60%. One can suppose that it was due to the oxidant depletion during the reaction run. However, the presence of H_2_O_2_ remaining in the post-reaction solutions was confirmed by a simple qualitative test (by applying potassium iodide and starch mixture). Nevertheless, to make sure that the initial amount of H_2_O_2_ was not the limiting factor affecting the catalytic performance, a test with the use of a larger amount of H_2_O_2_ (3.0:1.0 oxidant to glucose molar ratio) for a chosen catalyst (Au/HBeta) was performed. It was established that the increase in H_2_O_2_/glucose molar ratio from 2.2 to 3.0 did not result in any significant enhancement of glucose conversion (49.1 vs. 50.8%, respectively). It was therefore decided to keep the H_2_O_2_/glucose molar ratio of 2.2 in all the experiments.

The mechanism of microwave-assisted glucose oxidation in the presence of hydrogen peroxide has not been yet clearly elucidated and current literature does not provide exhaustive answers to the questions about possible reaction pathways in the glucose oxidation reaction. Rautiainen et al. [50] have shown a correlation between the rate of oxygen formation and the activity of gold catalysts. The most active Au/Al_2_O_3_ exhibited the highest rate of H_2_O_2_ decomposition towards O_2_ formation. Bearing in mind that the differences in glucose conversion between the calcined and non-calcined gold zeolites were negligible when using hydrogen peroxide as an oxidative agent, it is supposed that the molecular oxygen evolution resulting from hydrogen peroxide decomposition on Au NPs—rather than the main oxidation step of carbonyl group of a glucose molecule—could be a possible rate-limiting step in the overall process. Microwave-assisted oxidation of glucose with H_2_O_2_ is undoubtedly a promising alternative to the conventional oxidation of glucose with oxygen, but further research should be carried out to shed more light on the mechanism of hydrogen peroxide activation.

## 4. Conclusions

Different combinations in modification of the zeolite supports (APMS grafting on calcined and non-calcined gold zeolites as well as boron impregnation) for gold and the use of zeolites of different structures (two-dimensional MCM-36 and three-dimensional HBeta) allowed us to establish some important correlations between various parameters and the activity of gold catalysts in glucose oxidation with molecular oxygen and hydrogen peroxide. Much higher glucose conversion in oxidation with both, molecular oxygen and hydrogen peroxide, obtained in the presence of Au/MCM-36 and Au/NH_2_/MCM-36 than that got for Au/HBeta and Au/NH_2_/HBeta, respectively, indicated the role of the support porosity/structure/texture properties. The more open the structure (larger pores), the higher the glucose conversion. For the supports of the same structural/textural features (Au/B/MCM-36 and Au/MCM-36), the level of negative charge accumulation on metallic gold particles (a lower for the boron containing catalyst) determined chemisorption of molecular oxygen and decomposition of hydrogen peroxide, and in this way influenced glucose conversion (higher for the reaction performed in the presence of Au/MCM-36 with a higher electron density on gold particles). The role of gold NPs with partial negative charge on their surface was also confirmed by comparison of the activity of a single surface gold atom (expressed by TOF) for two catalysts containing Au NPs of the same average size of 7 nm, namely Au/HBeta (lower partial negative charge on gold particles) and Au/MCM-36 zeolites (higher partial negative charge on Au NPs). The first catalyst showed a much lower TOF value.

Non-calcined Au/NH_2_/zeolites contained the smallest Au NPs. The presence of APMS in non-calcined gold zeolites, independently of the support structure and composition, led to a significant growth of the glucose conversion in the oxidation with molecular oxygen and almost did not change their activity in the oxidation with hydrogen peroxide, although the gold particles were well dispersed. The amine groups, protonated during the procedure of gold anchoring, took part in the chemisorption of glucose, which enhanced the catalytic activity in glucose oxidation with oxygen. The presence of APMS in calcined gold zeolites (NH_2_/Au/zeolites) did not enhance their activity, which emphasizes the role of the protonated amine groups. As glucose chemisorption on the protonated amine species did not increase the glucose conversion in the oxidation with H_2_O_2_, one can conclude that in this case the reaction rate was not limited by sugar chemisorption but by decomposition of hydrogen peroxide on gold particles.

## Figures and Tables

**Table 1:** Composition, mean Au NPs sizes and textural properties of prepared catalysts.

Entry	Catalyst	%_wt._ Au ^a^	%_wt._ N ^b^	Au NPs Size ^c^, nm	BET Surface Area, m^2^ g^−1^	t-Plot External Surface Area m^2^ g^−1^	Single Point Total Pore Volume ^d^ cm^3^ g^−1^	t-Plot Micropore Volume, cm^3^ g^−1^
1.	HBeta	–	traces	–	526	149	0.34	0.20
2.	Au/HBeta	1.87	traces	7.0	338	82	0.24	0.14
3.	Au/NH_2_/HBeta	2.37	1.56	2.5	132	78	0.13	0.03
4.	NH_2_/Au/HBeta	1.78	1.74	6.6	57	25	0.04	0.02
5.	MCM-36	–	traces	–	534	361	0.54	0.10
6.	Au/MCM-36	1.84	traces	7.0	413	191	0.43	0.10
7.	Au/NH_2_/MCM-36	2.33	1.50	2.8	217	154	0.32	0.03
8.	NH_2_/Au/MCM-36	1.91	1.36	6.7	210	106	0.24	0.06
9.	B/MCM-36	–	traces	–	546	401	0.53	0.08
10.	Au/B/MCM-36	1.58	traces	5.3	401	241	0.41	0.09
11.	Au/NH_2_/B/MCM-36	1.55	1.87	3.2	108	38	0.21	0.02

^a^ Gold loadings were determined by ICP-OES. ^b^ The amount of nitrogen in the samples was examined by elemental analysis. ^c^ The mean gold particle sizes were calculated from TEM images. The calculations were performed on the basis of 200 particles. ^d^ The total pore volumes were evaluated at a relative pressure of 0.98.

**Table 2:** Content of Brønsted (BAS) and Lewis (LAS) acid sites occupied by pyridine after desorption at different temperatures. The calculation based on intensity of IR bands (1545 cm^−1^ for BAS and 1455 cm^−1^ for LAS) and extinction coefficients of 0.044 cm^2^ µmol^−1^ for BAS and 0.165 cm^2^ µmol^−1^ for LAS from [23].

Entry	Catalyst	Evacuation Temp., °C	Number of BAS Occupied by Pyridine, after Evacuation, μmol g^−^^1^	Number of LAS Occupied by Pyridine after Evacuation at 300 °C, μmol g^−^^1^	Pyridine Desorbed at 300 °C from BAS, % ^a^	BAS/LAS Ratio After Evacuation at 300 °C
1.	HBeta	150	434	–	34.3	1.54
		200	452	–		
		250	360	–		
		300	297	193		
2.	Au/HBeta	150	344	–	44.8	1.49
		200	348	–		
		250	244	–		
		300	192	129		
3.	MCM-36	150	425	–	22.4	5.73
		200	406	–		
		250	373	–		
		300	315	55		
4.	Au/MCM-36	150	188	–	24.7	3.77
		200	150	–		
		250	148	–		
		300	113	30		
5.	Au/B/MCM-36	150	198	–	35.2	4.07
		200	176	–		
		250	150	–		
		300	114	28		
6.	B/MCM-36	150	482	–	5.3	6.31
		200	360	–		
		250	328	–		
		300	341	54		

^a^ Related to the amount of pyridine chemisorbed after evacuation at 200 °C.

**Table 3:** Results of base-free oxidation of glucose with molecular oxygen over selected catalysts.

Entry	Catalyst	Glucose Conv. ^a^, %	Selectivity, %		TOF ^b^, h^−1^
			A	B	
1.	HBeta	1.2	82.0	18.0	–
2.	Au/HBeta	50.6	98.8	1.2	24,200
3.	Au/NH_2_/HBeta	60.7	99.2	0.8	10,400
4.	NH_2_/Au/HBeta	12.0	>99.9	traces	5410
5.	MCM-36	6.2	89.0	11.0	–
6.	Au/MCM-36	65.2	99.2	0.8	31,300
7.	Au/NH_2_/MCM-36	77.5	99.3	0.7	14,800
8.	NH_2_/Au/MCM-36	61.0	99.1	0.9	28,000
9.	B/MCM-36	<1	>99.9	traces	–
10.	Au/B/MCM-36	45.8	98.9	1.1	16,600
11.	Au/NH_2_/B/MCM-36	68.1	99.1	0.9	14,900

^a^ Reaction conditions: 20 mL of 0.2 M glucose solution, glucose/Au (molar ratio) = 1970/1, mixing rate = 600 rpm, T = 110 °C, pO_2_ = 0.5 MPa, time = 1 h; A–gluconic acid, B–glucuronic acid. ^b^ The number of moles of gold atoms localized on the external surface of spherical Au particles was taken into account in the TOF calculations: (the number of moles of glucose converted after 1 h) × (the number of moles of gold atoms localized on the external surface of the Au NPs in a given mass of the catalyst)^−1^ × h^−1^; based on Au NPs size calculated from TEM.

**Table 4:** Comparison of TOF values of different gold catalysts in base-free glucose oxidation with molecular oxygen as oxidant.

Entry	Catalyst Symbol	%_wt._ Au ^a^	Au NPs Size, nm	Reaction Conditions	TOF, h^−1^	Reference
1.	Au/MCM-36	1.84	7.0	20 mL of 0.2 M glucose solution, glucose/Au molar ratio = 1970, T = 110 °C, p(O_2_) = 0.5 MPa	31,300 ^a^	This work
2.	Au/TH-150	0.5	1.2	10 mL of 0.1 M glucose solution, glucose/Au molar ratio = 1000, T = 110 °C, p(O_2_) = 1 MPa	1920 ^b^	[4]
3.	Au/CMK-3	0.94	3.0	20 mL of 0.1 M glucose solution, glucose/Au molar ratio = 1000, T = 110 °C, p(O_2_) = 0.3 MPa	17,700 ^c^	[46]
4.	Au-HBeta(AP)	1.4	6.0	20 mL of 0.2 M glucose solution, glucose/Au molar ratio = 1970, T = 110 °C, p(O_2_) = 0.5 MPa	26,500 ^d^	[17]
5.	Au/SBA-15	2.1	4.9	10 mL of 0.2 M glucose solution glucose/Au molar ratio = 1970, T = 110 °C, p(O_2_) = 0.5 MPa	16,900 ^d^	[47]
6.	Au/HAP-LDH	0.22	6.6	12 mL of 0.167 M glucose solution glucose/Au molar ratio = 1000, T = 110 °C, p(O_2_) = 0.5 MPa	20,200 ^e^	[48]

Calculated after: ^a^ 60 min of the reaction; ^b^ 15 min of the reaction; ^c^ 5 min of the reaction; ^d.^120 min of the reaction; ^e^ calculated for low conversions (<30%).

**Table 5:** Results of microwave-assisted oxidation of glucose with hydrogen peroxide over selected catalysts.

Entry	Catalyst	Glucose Conv. ^a^, %	Selectivity, %		TOF ^b^, h^−1^
			A	B	
1.	HBeta	13.3	93.9	6.1	
2.	Au/HBeta	49.1	99.0	1.0	158,000
3.	Au/NH_2_/HBeta	44.4	98.9	1.1	50,900
4.	NH_2_/Au/HBeta	12.2	95.2	4.8	37,100
5.	MCM-36	3.8	83.6	16.4	–
6.	Au/MCM-36	59.2	99.2	0.8	191,000
7.	Au/NH_2_/MCM-36	59.5	99.2	0.8	76,500
8.	NH_2_/Au/MCM-36	45.9	99.0	1.0	142,000
9.	B/MCM-36	7.2	72.2	27.7	–
10.	Au/B/MCM-36	49.5	99.0	1.0	121,000
11.	Au/NH_2_/B/MCM-36	56.6	99.1	0.9	83,800

^a^ Reaction conditions: glucose/Au (molar ratio) = 1970/1, 8 mL of 0.2 M glucose solution, T = 110 °C, 2.2 equiv. H_2_O_2_, time = 10 min.; A–gluconic acid, B–glucuronic acid, ^b^ The number of moles of gold atoms localized on the external surface of spherical Au particles was taken into account in the TOF calculations: (the number of moles of glucose converted after 1 h) × (the number of moles of gold atoms localized on the external surface of the Au NPs in a given mass of the catalyst)^−1^ × h^−1^; based on Au NPs size calculated from TEM.

## Data Availability

The data presented in this study are available on request from the corresponding authors via e-mail: adrian.walkowiak@amu.edu.pl (A.W.), j.wolska@amu.edu.pl (J.W.).

## Supplementary Materials

The following are available online at https://www.mdpi.com/article/10.3390/ma14185250/s1, Figure S1: ^11^B MAS NMR spectrum of B/MCM-36 sample, Figure S2: FTIR spectra after (a) adsorption of pyridine at 150 °C and desorption at (b) 150 °C, (c) 200 °C, (d) 250 °C, (e) 300 °C. All the spectra were obtained by subtraction the spectrum after activation and normalized to the density of a wafer of ca 10 mg cm^−2^, Figure S3: Representative TEM image of Au/HBeta showing Au NPs localization on the external surface of the zeolite, Figure S4: ATR-FTIR spectra of selected materials before and after glucose solution treatment and drying at 80 °C, Figure S5: N 1s region of XP spectra of gold-containing zeolites for (A) HBeta; (B) MCM-36 series. The regions of BE values typical to the protonated and non-protonated amine groups are marked in yellow and blue, respectively.
