# Supplementary material for: Modification of Gold Zeolitic Supports for Catalytic Oxidation of Glucose to Gluconic Acid
Source: Materials (Basel). 2021 Sep 13;14(18):5250. doi: 10.3390/ma14185250 (PMC8467280; doi:10.3390/ma14185250)
Supplement: Supplementary file 1 [file materials-14-05250-s001.zip › materials-1315018-supplementary.pdf]

Supplementary Materials

for Materials

Modification of gold zeolitic supports for catalytic oxidation of glucose to gluconic acid

Adrian Walkowiak*, Joanna Wolska*, Anna Wojtaszek-Gurdak, Izabela Sobczak, Lukasz Wolski, Maria Ziolek

Adam Mickiewicz University, Poznań, Faculty of Chemistry, Uniwersytetu Poznańskiego 8, 61-614 Poznań, Poland

* *corresponding author*

e-mail: adrian.walkowiak@amu.edu.pl (A.W.) ; j.wolska@amu.edu.pl (J.W.)

List of content:

Figure S1. ^{11}B MAS NMR spectrum of B/MCM-36 sample.

Figure S2. FTIR spectra after (a) adsorption of pyridine at 150 °C and desorption at (b) 150 °C, (c) 200 °C, (d) 250 °C, (e) 300 °C. All the spectra were obtained by subtraction the spectrum after activation and normalized to the density of a wafer of ca 10 mg cm^{-2}.

Figure S3. Representative TEM image of Au/HBeta showing Au NPs localization on the external surface of the zeolite.

Figure S4. ATR-FTIR spectra of selected materials before and after glucose solution treatment and drying at 80°C.

Figure S5. N 1s region of XP spectra of gold-containing zeolites for (A) HBeta; (B) MCM-36 series. The regions of BE values typical to the protonated and non-protonated amine groups are marked in yellow and blue, respectively.

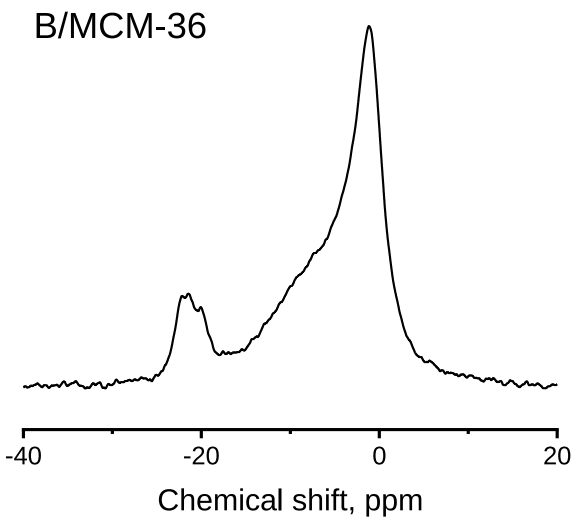

Figure S1. ^{11}B MAS NMR spectrum of B/MCM-36 sample.

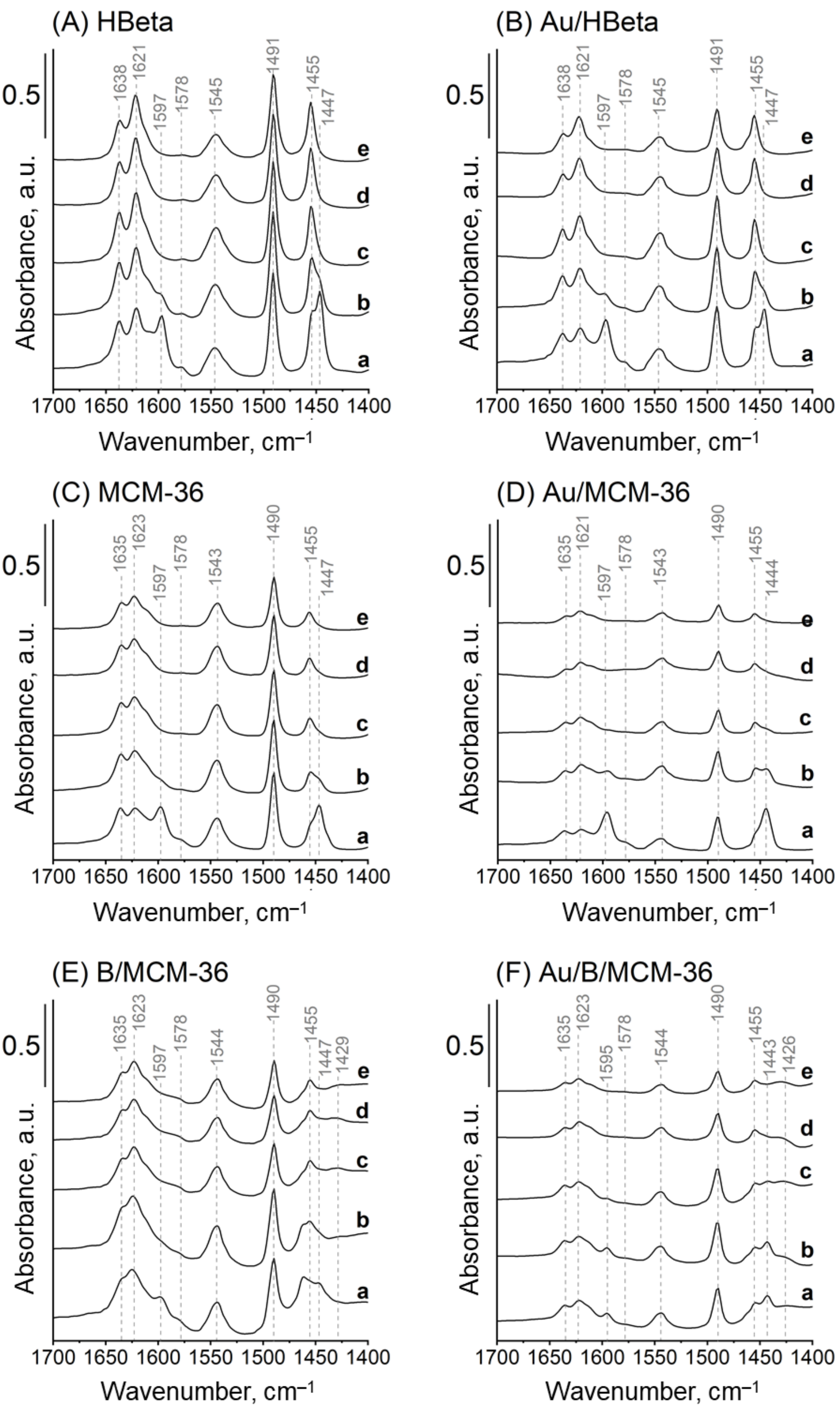

Figure S2. FTIR spectra after (a) adsorption of pyridine at 150 °C and desorption at (b) 150 °C, (c) 200 °C, (d) 250 °C, (e) 300 °C. All the spectra were obtained by subtraction the spectrum after activation and normalized to the density of a wafer of 10 mg cm^{-2}.

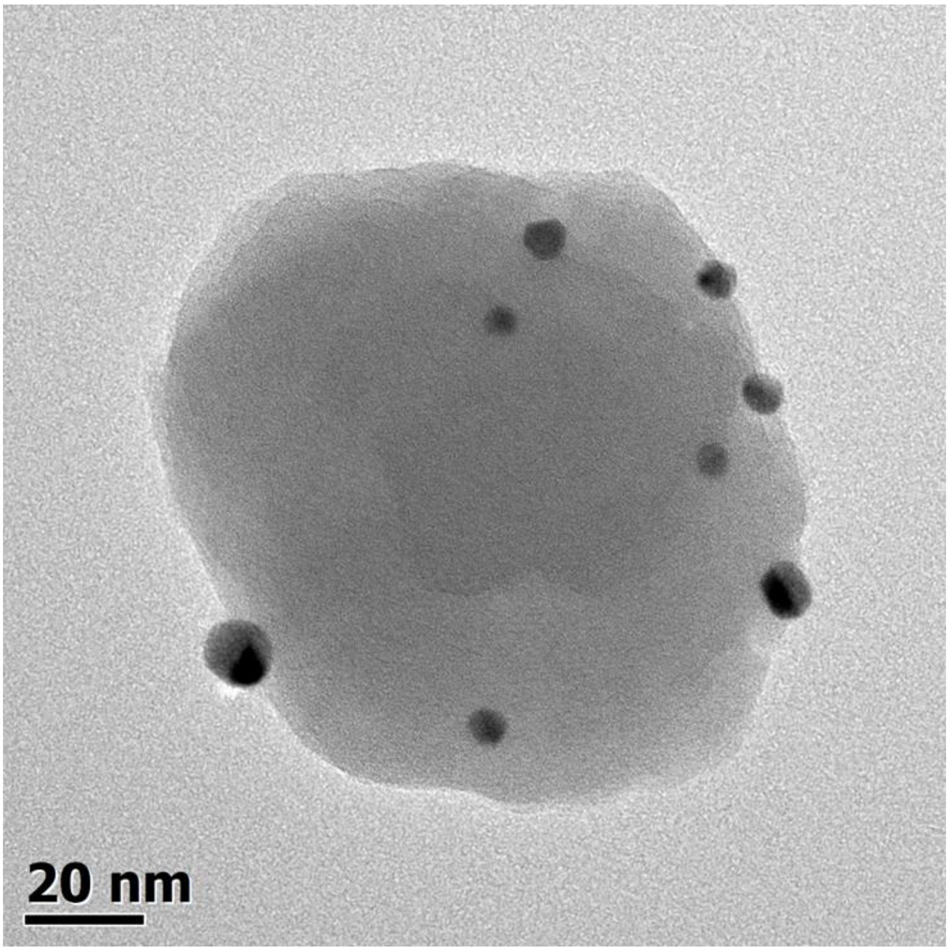

Figure S3. Representative TEM image of Au/HBeta showing Au NPs localization on the external surface of the zeolite grain.

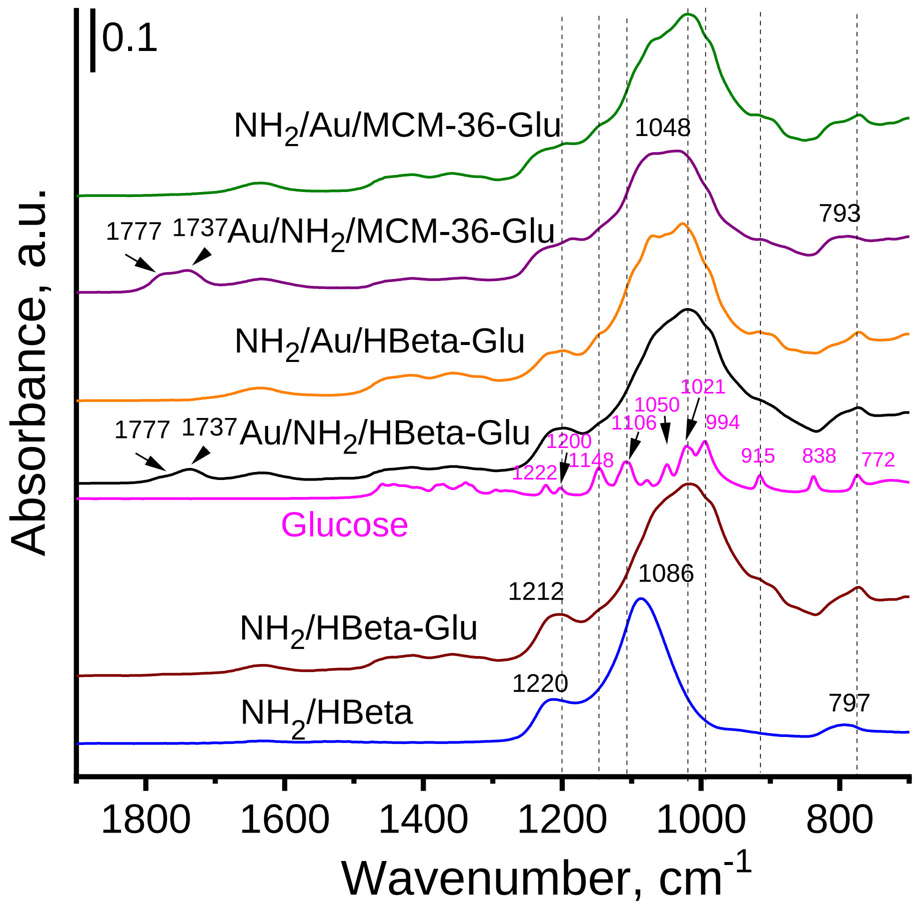

Figure S4. ATR-FTIR spectra of selected materials before and after glucose solution treatment and drying at 80°C.

Adnotation: "-Glu" in the catalyst symbol means sample after glucose adsorption and drying.

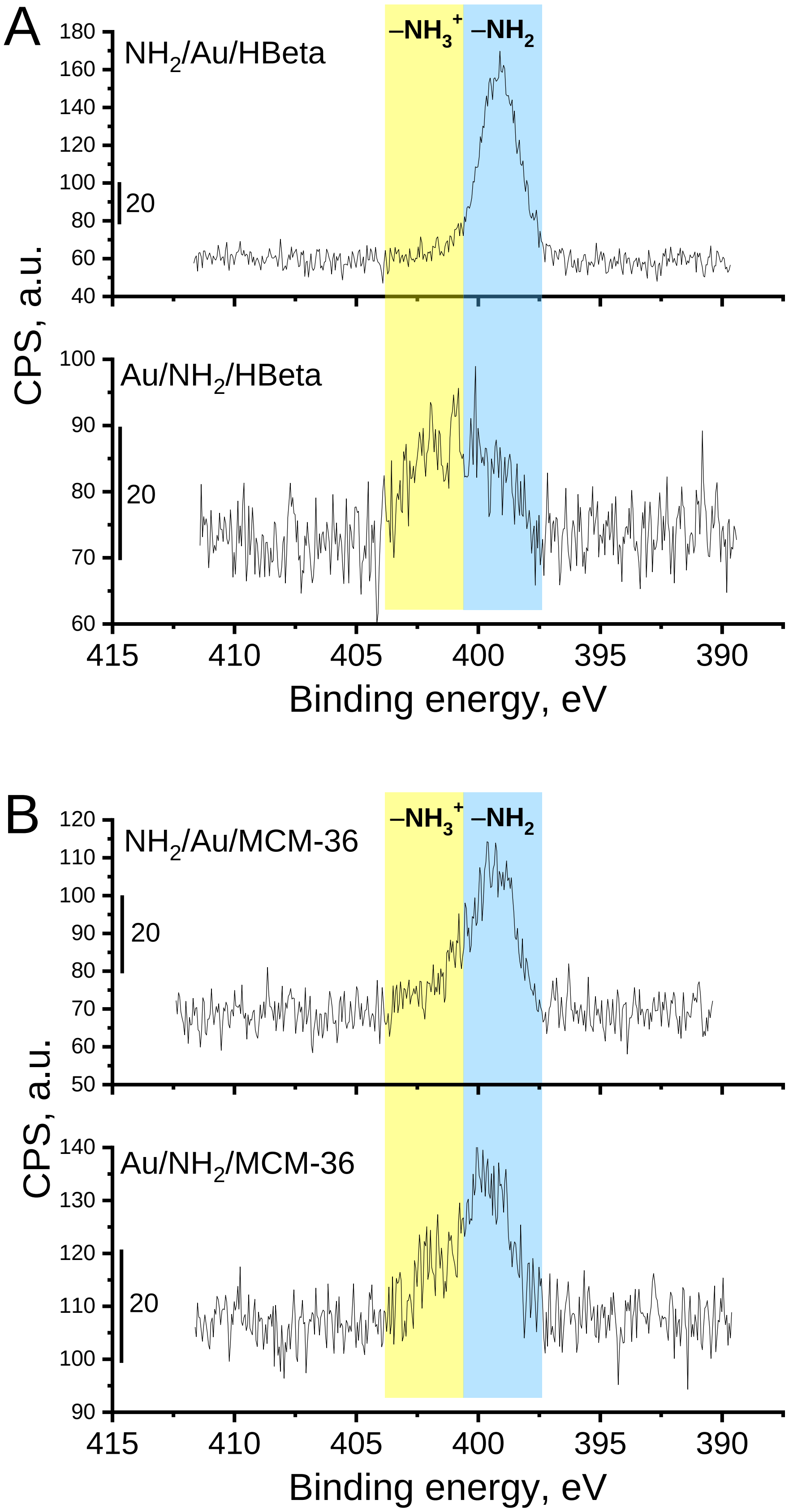

Figure S5. N 1s region of XP spectra of gold-containing zeolites for (A) HBeta; (B) MCM-36 series. The regions of BE values typical to the protonated and non-protonated amine groups [Graf et al. *Surf. Sci.* **2009**, *603*, 2849–2860, doi:10.1016/j.susc.2009.07.029] are marked in yellow and blue, respectively.
